# Origin of Multiferroism in VOX_2_ (X = Cl, Br, I) Monolayers

**DOI:** 10.3390/nano14050408

**Published:** 2024-02-23

**Authors:** Angel Todorov Apostolov, Iliana Naumova Apostolova, Julia Mihailova Wesselinowa

**Affiliations:** 1 University of Architecture Civil Engineering and Geodesy, Hristo Smirnenski Blvd. 1, 1046 Sofia, Bulgaria; 2 University of Forestry, Kl. Ohridsky Blvd. 10, 1756 Sofia, Bulgaria; inaapos@abv.bg; 3 Faculty of Physics, Sofia University “St. Kliment Ohridski”, J. Bouchier Blvd. 5, 1164 Sofia, Bulgaria

**Keywords:** multiferroism, magnetoelectric interaction, two-dimensional system, spin-reorientation transition

## Abstract

Based on the proposed microscopic model, we investigate the multiferroic characteristics of VOX_2_ (X = Cl, Br, I) monolayers using a Green’s function method. The dependence of the microscopic parameters of the ferroelectric system (pseudo-spin arrangement and flipping rate) on the magnitude and sign of the exchange magnetic interaction along the *b*-axis and the value of the Dzyaloshinskii–Moria vector have been investigated and qualitatively explained. The possibility of observing a spin-reorientation transition with a change in the character of spin ordering from antiferromagnetic to ferromagnetic is investigated. It is found that the antisymmetric magnetoelectric interaction may be responsible for the spin-reorientation transition without a change in the ordering of magnetic moments. Changing the sign of the exchange magnetic interaction along the *b*-axis leads to ferromagnetic ordering without observing a spin-reorientation transition. The dependence of isotropic and antisymmetric magnetic interactions on the microscopic parameters of the ferroelectric system is qualitatively explained. A mechanism for the occurrence of the spin-reorientation transition is presented based on the proposed microscopic model. The obtained results qualitatively coincide with Density Functional Theory calculations.

## 1. Introduction

Over the last decade, significant strides have been made in uncovering 2D crystals that exhibit magnetic ordering [1,2,3,4]. These materials have attracted attention due to their potential practical applications and the prospect of downsizing memory and logical devices. At the same time, there have been descriptions of monolayers and ultra-thin films, composed of multiple layers, that showcase ferroelectric properties at room temperatures [5,6]. A contemporary challenge for physics is the synthesis or prediction of 2D materials (monolayers and ultra-thin films) with multiferroic properties, where both magnetism and polarization are observed in a single phase. Recently, the presence of such 2D materials has been predicted, including ferromagnetic–ferroelectric electronically doped CrBr_3_ [7] and ferromagnetic–antiferroelectric monolayer transition metal phosphorus chalcogenides [8], which are proper (type I) 2D multiferroics. Hf_2_VC_2_ F_2_ [9] is a monolayer with y Y-type noncollinear spin structure, i.e., unproper multiferroic (type II).

Multiferroics (MFs) are substances that simultaneously exhibit various ferroic orderings: magnetic ordering (ferromagnetism, antiferromagnetism, ferrimagnetism, spiral structures), electric ordering (ferroelectricity, antiferroelectricity), and/or ferroelasticity in a single phase [10,11,12,13,14]. The connection between the electric and magnetic ordering parameters is determined by the magnetoelectric (ME) interaction. The occurrence of both magnetic and ferroelectric phase transitions in MFs makes them rare in nature. The reason for this is the so-called “*d*^0^ rule” [15] or “*d*^0^ – *d^n^* problem”. This rule states that ferroelectricity in transition metal oxides is observed only when the transition metal ions have empty d orbitals (“$d^{0}$ rule”), whereas for magnetism the transition metal ion must have partially filled d orbitals. From a theoretical standpoint, if there is one electron in the d orbital, it resides in a hybridized state. This does not favour the formation of a coordinate bond and leads to the destabilization of the ferroelectric phase.

The microscopic origin of magnetism is uniform across all MFs. However, the scenario with ferroelectricity is notably different. There exist several microscopic sources of polarization, defining various types of MFs—proper (Type I) and unproper (Type II). 

In Type I MFs, ferroelectricity and magnetism stem from different origins, often resulting from distinct active “subsystems” within the elementary cell. In other words, polarization and magnetization are attributed to different functional units. This leads to considerably different phase transition temperatures in the two subsystems [16], and the correlation between the two ordering parameters is weak.

In Type II MFs, ferroelectricity is induced by spin ordering. Dipoles in this case are highly responsive to externally applied magnetic fields. While the ME coupling is robust in Type II MFs, the temperature of the phase transition where MF properties emerge is typically far from room temperature, limiting their practical applications [17].

In 2019, it was theoretically predicted [18,19] that a monolayer sample of VOX2 (X = Cl, Br, I) demonstrates multiferroic (MF) properties, similar to those observed in BaTiO_3_, which are crucial for proper ferroelectric behaviour. In VOX_2_ (X = Cl, Br), simultaneous antiferromagnetic (AFM) and ferroelectric (FE) ordering are observed, whereas in VOI_2_, ferromagnetic (FM) and FE ordering are presented. The ferroelectricity arises from the displacement of V ions from their centrosymmetric positions, causing the V-O bonds to become inequivalent. This results in a spontaneous polarization $P_{S}$ with magnitudes of *P_S_*(VOCl_2_) = $91\frac{\mu C}{{cm}^{2}}$, *P_S_*(VOBr_2_) = $75\frac{\mu C}{{cm}^{2}}$ and *P_S_*(VOI_2_) = $61\frac{\mu C}{{cm}^{2}}$. These values are comparable to the spontaneous polarization of proper MFs (e.g., BiFeO_3_) [20,21,22] and larger than those in unproper MFs [23].

Based on Density Functional Theory (DFT) [18], it has been demonstrated that the barrier height of the double-well potential in a monolayer of VOX_2_ is similar to the height observed in typical ferroelectrics [24,25]. Consequently, the temperature of the FE phase transition is expected to be higher than room temperature [26,27]. This unequivocally indicates that the FE behaviour in the monolayer is a similar to materials that perform the “$d^{0}$ rule”, despite V^4+^ having a $d^{1}$ configuration and a spin S=1/2. The electron occupies the lowest-energy $d_{xy}$ orbital, situated in the plane perpendicular to the V-O bond, and according to [28], the coupling between the $d_{xy}$ orbital of the V cation and the *p* orbital of the O anion is zero. Thus, this electron does not hinder the formation of a stable covalent bond and does not destabilize the FE phase. The magnetism in the monolayer arises from the unpaired electron in the $d_{xy}$ orbital of the V^4+^ ion, resulting in a local magnetic moment of 1μ*_B_*/V.

Using the generalized gradient approximation (GGA) in [18,19], it is demonstrated that the primary contribution to the AFM ordering of magnetic moments in VOX_2_ (X = Cl, Br) comes from the direct exchange interaction, which is inherently AFM in nature. This exchange interaction prevails over the superexchange interaction in the V-X-V bond, which exhibits FM characteristics. This is the reason why the magnetic moments in the monolayer are arranged antiparallel to the nearest neighbors and parallel to the second neighbours. In VOI_2_, the direct exchange interaction is comparatively smaller than the superexchange interaction, resulting in FM ordering of the magnetic moments [19]. Monte Carlo simulations determine transition temperatures to the magnetically ordered state ranging from 177 K in VOCl_2_ [18] to 21 K in VOI_2_ [29]. For all VOX_2_ compounds, polarization is perpendicular to magnetization.

Remarkably, in these monolayers, the V ion plays a crucial role in both FE and magnetic ordering. On one hand, the emergence of spontaneous polarization categorizes VOX_2_ as a Type I MFs. The overlap between the $d_{xy}$ orbitals of V ions and their hybridization with the p orbitals of X cations determine the nature of the magnetic ordering. According to [18,19], this leads to an additional modification of polarization, inherent to Type II multiferroics. Thus, in the monolayers of VOX_2_ (X = Cl, Br, I), properties of both Type I and Type II MFs naturally combine. The mechanism of this combination remains unclear, as well as the nature of the ME coupling between the two ordering parameters. Unfortunately, experimental results providing additional information on polarization and magnetization in these samples, along with their dependence on external magnetic and electric fields, validating their multiferroic nature, are currently lacking.

The aim of the present publication is to explore the MF properties of VOCl_2_. Using a microscopic model, the influence of the fundamental interactions and applied external magnetic and electric fields on polarization and magnetization is investigated.

## 2. Materials and Method

**Figure 1:**
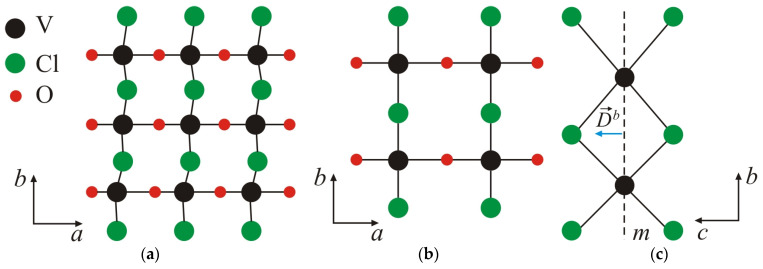
(**a**) Polar FE structure (*Pmm*2) of the VOCl_2_ monolayer; (**b**) centrosymmetric PE structure of the VOCl_2_ monolayer; (**c**) orientation of the DM vector ${\vec{D}}_{ij}^{b}$, determining the antisymmetric interaction in the V-Cl-V bond. The dashed line indicates the mirror plane of symmetry *m*.

The crystal structure of the monolayer (2D) material with the structural formula VOCl_2_ is orthorhombic with a *Pmm*2 symmetry group. The V ion is bonded to four Cl and two O atoms (Figure 1), creating a polar structure without an inversion centre. The V ion experiences displacement from the centre of the VO_2_Cl_4_ octahedron along the $a\left(x\right)$-axis (Figure 1a). Consequently, the two neighbouring V-O bonds become inequivalent, giving rise to spontaneous polarization along the a(x)-axis. This behaviour is also observed in bulk samples [30]. The V-Cl bonds remain equivalent, and only the angle of the O-V-Cl bond deviates from 90°. As the temperature increases, a structural phase transition from the FE to the paraelectric (PE) state occurs. In this transition, the lengths of the corresponding V-O and V-Cl bonds become equal, resulting in a centrosymmetric structure (Figure 1b). This transition is of the displacive type, typical to those observed in ABO_3_ compounds. Theoretical numerical calculations [31] estimate the FE switching value to be around 0.18 eV/f.u. and his value significantly exceeds the energy of thermal fluctuations at room temperature [32], indicating the stability of the FE phase at these temperatures. Furthermore, the decrease in symmetry leads to a decrease in total energy, as the value of the depth of two-atom is close to that of typical FE materials such as BiFeO_3_, PbTiO_3_ and LiNbO_3_ [25,33].

**Figure 2:**
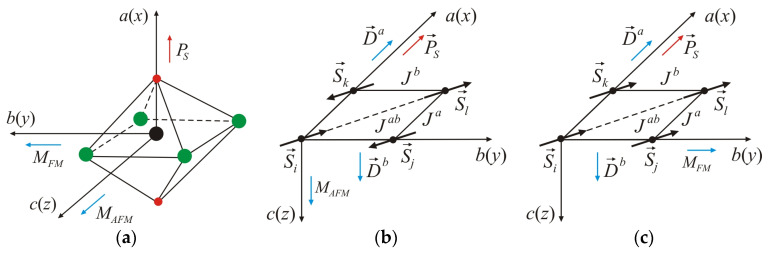
(**a**) VO_2_Cl_4_ octahedron, where *a*, *b*, *c* are the lattice parameters along the *x*-, *y*-, and *z*-axes, respectively. *c*(*z*)-axis determines the axis of easiest magnetization in AFM ordering of magnetic moments, *b*(*y*)-axis is the axis of easiest magnetization in FM ordering of spins, and *a*(*x*) determines the direction of spontaneous polarization *P_S_*; (**b**) antiparallel ordering of magnetic moments in (*ab*) plane with indicated possible isotropic and antisymmetric exchange interactions, and orientations of magnetization *M* and polarization *P_S_* in the monolayer; (**c**) parallel ordering of magnetic moments in (*ab*) plane with indicated possible isotropic and antisymmetric interactions, and orientations of magnetization *M* and polarization *P_S_* in the monolayer.

This allows for the possibility to determine the polarization properties of the VOCl_2_ monolayer using the transverse Ising model (TIM) in a pseudo-spin representation proposed by Blinc and de Gennes, which describes order–disorder and displacive-type ferroelectrics [33,34]. The spontaneous polarization $P_{S}$ is along the a(x)-axis. When choosing the coordinate system, as shown in Figure 2a, the Hamiltonian takes the form:

(1) $$H_{e}=-\Omega \sum_{i}B_{i}^{y}-\frac{1}{2}\sum_{ij}J_{ij}^{\prime }B_{i}^{x}B_{j}^{x}-\varepsilon \vec{E}\cdot \sum_{ij}{\vec{B}}_{i}.$$

The pseudo-spin operator $B_{i}^{x}$ represents the two positions of the ferroelectric unit at the lattice site *i*. $J_{ij}^{\prime }$ signifies the pseudo-spin interaction. $\vec{E}$ is the external electric field. The dynamics of the FE parts are implemented by the first term involving the flipping rate Ω and the operator $B_{i}^{y}$. It must be noted that with the Green’s function method, the transverse Ising model can be used for theoretical and numerical calculations of temperature dependencies of static and dynamic properties of ferroelectrics over a wide range of temperatures. The transverse Ising model in its pseudo-spin representation allows for avoidance of strong anharmonicity. The finite number of spin states simplifies the problem to such an extent that it goes beyond the scope of standard perturbation theories and mean-field methods.

**Figure 3:**
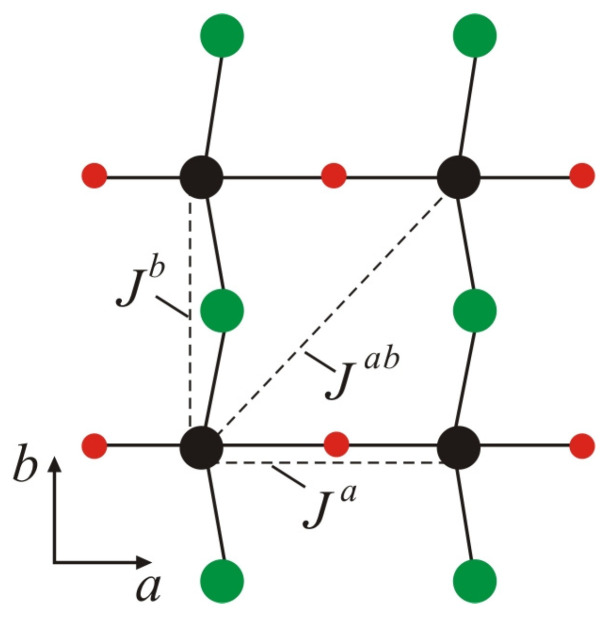
Schematic representation of exchange interactions between nearest neighbours *J^a^* and *J^b^* along the *a*- and *b*-axes, respectively, and the exchange interaction *J^ab^* between second neighbors. *J^b^* determines the resultant interaction between spins associated with direct V…V exchange and superexchange in the V-Cl-V bond along the *b*-axis.

The magnetism in the VOCl_2_ monolayer is governed by the spin of the non-paired electron in the $d_{xy}$ orbital of the V^4+^ ion, resulting in a local magnetic moment of 1μ*_B_*. DFT calculations indicate that the magnetic ordering is AFM (Figure 2b), where magnetic moments are arranged antiparallel along the a- and b-axes. The superexchange interaction between the spins of V ions along the b-axis, assisted by Cl anions, is weak according to the Goobenough–Kanamori–Ardenson rules [35] (electron transfer at a V-Cl-V bond angle close to 90° is restricted by symmetry considerations (Figure 3)). A similar situation is observed for the superexchange interaction in the V-O-V bond along the a-axis (Figure 3), which is weak due to lack of overlap (or weak overlap) between the p orbitals of O ions and the $d_{xy}$ orbitals of V cations [36]. Along the b-axis, the superexchange interaction V-Cl-V should be align the magnetic moments of V ions in a parallel (FM) configuration. On the contrary, in [37], it has been reported that these substances exhibit direct exchange interaction between V spins along the b-axis, which is AFM (attributed to the short distance between spins along this axis b=3.380 Å [19]). Due to the large distance between the spins along the a-axis (a=3.799 Å [19]), such interaction is negligibly small and is FM. The interactions along the a- and b-axes will be denoted as $J^{a}$ and $J^{b}$, respectively. These are interactions between nearest neighbours (Figure 3). The direct V…V interaction along the diagonal $J^{ab}$ (Figure 3) is also negligibly small, which is FM and determines the coupling between second neighbours. It should be noted that along the b-axis, there is competition between direct AFM and FM superexchange in the V-Cl-V bond. The first prevails over the second, and under $J^{b}$, we will understand the effective interaction resulting from both.

In 2D systems, taking into account magnetic anisotropy is essential to stabilize long-range spin ordering [38,39]. Based on DFT calculations incorporating spin–orbit coupling [36], it has been proven that the easy magnetization axis for VOCl_2_ is in the [0,0,1] direction, parallel to the c(z)-axis (Figure 2a,b). This means that in the monolayer structure, magnetization is perpendicular to the layer. It is important to note that applying tensile stress along the b-axis induces a transition from AFM to FM ordering with a reorientation of magnetization along the b(y)-axis ([0,1,0] direction). This transition represents a spin-reorientation (SR), wherein the spontaneous polarization $P_{S}$ and magnetization M lie in the plane of the monolayer, remaining mutually perpendicular. This indicates the presence of an easy magnetization axis along the b(y)-axis (Figure 2a,c).

The breaking of spatial inversion symmetry in the VOCl_2_ monolayer due to the displacement of V ions from their centrosymmetric positions may lead to the emergence of antisymmetric exchange interactions of the Dzyaloshinskii–Moriya (DM) type [40,41]. This type of interaction depends on the DM vector (denoted as $\overset{⃑}{D}$). Following the rules that determine the influence of individual symmetry elements on the direction and magnitude of the DM vector, as defined in [40,42], for the bending V-Cl-V bond along the b-axis, the DM vector is expected to be oriented along the c(z)-axis (Figure 2b). This is determined by the requirement that this vector must be perpendicular to the mirror plane of symmetry (m), which passes through the positions of V ions (Figure 1c). From symmetry considerations, it is clear that antisymmetric interaction for a V-O-V bond is forbidden. In the absence of inversion symmetry for FE phase and following the selection rules in [41], antisymmetric exchange interaction can also be established between second neighbours (Figure 3). For that case, the DM vector will have a component only along the a(x)-axis (Figure 2b). DFT calculations [29] show that the latter antisymmetric interaction has a very small value of the DM vector (below 0.01 meV) and can be neglected in further numerical calculations.

It should be noted that the inclusion of antisymmetric DM interaction in the description of the magnetic properties of the system is due to the observed SR transition from AFM to FM ordering, with a change in the magnetization direction from out-of-plane (c(z)-axis) to in-plane (b(y)-axis). From a theoretical perspective, this transition is determined by a direction-dependent interaction, such as the DM interaction. From a theoretical point of view, this transition is determined by an interaction depending on the direction, such as the DM interaction.

Based on the above discussion, the Hamiltonian describing the magnetic properties of the VOCl_2_ monolayer takes the following form:

(2) $$H_{m}=-\sum_{{\langle ij\rangle }_{NNb}}J_{ij}^{b}{\vec{S}}_{i}\cdot {\vec{S}}_{j}-\sum_{{\langle ik\rangle }_{NNa}}J_{ik}^{a}{\vec{S}}_{i}\cdot {\vec{S}}_{k}-\sum_{{\langle il\rangle }_{NNN}}J_{il}^{ab}{\vec{S}}_{i}\cdot {\vec{S}}_{l}-\sum_{{\langle ij\rangle }_{NNb}}{\vec{D}}_{ij}^{b}\cdot [{\vec{S}}_{i}\times {\vec{S}}_{j}]-\;-\sum_{i}K^{c\left(z\right)}{\left(S_{i}^{z}\right)}^{2}-\sum_{i}K^{b\left(y\right)}{\left(S_{i}^{y}\right)}^{2},$$

where $J_{ij}^{b}$, $J_{ij}^{a}$ and $J_{il}^{ab}$ are the exchange interactions between the magnetic moments of the nearest neighbours along the b- and a- axes, respectively, $J_{il}^{ab}$ is between the second neighbors. $J_{ij}^{b}<0$—corresponds to AFM order, $J_{ij}^{a}>0$ and $J_{il}^{ab}$ > 0—correspond to FM order, where the following inequality holds: $\left|J_{ij}^{b}\right|\gg J_{il}^{ab}>J_{ij}^{a}$. It should be emphasized again that $J_{ij}^{b}$ is an effective interaction along the b-axis due to the competition between direct spin coupling and superexchange interaction assisted by Cl anions. The reason for the AFM arrangement of spins along the a-axis (although $J_{ij}^{a}>0\;$ (Figure 2b) is that the exchange interaction between the second neighbours is larger than the interaction between the nearest neighbours along the a-axis). ${\vec{D}}_{ij}^{b}$ is the DM vector characterizing the antisymmetric interaction between spins along the b-axis. As mentioned above it has the components $\left[0,\;0,\;d^{z}\right].$ This interaction is responsible for the appearance of a spiral structure that rotates clockwise in the (ab) plane. The vector of the cycloid is along the b-axis with a period of 47 lattice constants, i.e., 47*b* [29]. $K^{c\left(z\right)}$ and $K^{b\left(y\right)}$ are the constants of the single-ion magnetic anisotropy as $K^{c\left(z\right)}>K^{b\left(y\right)}$, and determining the c(z)- and b(y)-axes as the easy-axes of magnetization (Figure 2a).

The significant difference in the temperatures of the FE and magnetic phase transitions, $T^{FE}$ and $T^{AFM(FM)}$, characterizes the VOCl_2_ monolayer as a Type I MFs. The displacement of the V ion from its centrosymmetric position in the octahedra determines not only the FE arrangement but also the isotropic and antisymmetric interactions in the plane of the monolayer of VOCl_2_, associated with its nonzero magnetic moment and the overlap of the $d_{xy}$ orbitals. This feature establishes a correlation between the two ordering parameters, characteristic of Type II MFs.

On the other hand, it is well known that isotropic and antisymmetric exchange magnetic interactions strongly depend on changes in the length and angle of the V-Cl(O)-V bonds. The relative displacement of V ions concerning O ions along the a-axis will modulate these exchange interactions. If we denote the displacement of V ions from their equilibrium positions in the paraelectric region as “u” and expand the DM vector in a series with respect to these displacements, we obtain:

(3) $$\sum_{<ij>NNb}{\vec{D}}_{ij}^{b}\cdot \left({\vec{S}}_{i}\times {\vec{S}}_{j}\right)\approx \sum_{<ij>NNb}{\vec{D}}_{ij}^{b^{\prime }0}\cdot \left({\vec{S}}_{i}\times {\vec{S}}_{j}\right)+\lambda^{*}\sum_{<ij>NNb}(\vec{P_{S}}\times {\vec{e}}_{ij}^{b})\cdot \left({\vec{S}}_{i}\times {\vec{S}}_{j}\right),$$

where $\lambda^{*}=\lambda /e^{*}$, λ is the spin–lattice interaction resulting from relativistic spin–orbit coupling, $e^{*}$ is the Born effective charge, and $\left|\vec{P_{S}}\right|=e^{*}\langle u\rangle \;$ represents the spontaneous polarization. In the pseudospin representation, $\vec{P_{S}}=e^{*}\left[\;\frac{1}{N}\sum_{i}\langle B_{i}^{x}\rangle ,\;\frac{1}{N}\sum_{i}\langle B_{i}^{y}\rangle ,\;0\right]$. The last term in Equation (3) is crucial as it determines the mutual orientation of polarization and magnetic moment for which the total energy of the system $E_{tot}$ is minimized. In the case where polarization is perpendicular to the spins, the total energy reaches a minimum (this is the situation in the VOCl_2_ monolayer). This term defines the ME interaction, which depends on the direction. A similar mechanism has been defined and studied by Fishman et al. [43]. This term is formally similar to the spin–phonon interaction of the Peierls type. ${\vec{P}}_{S}$ is not a consequence of the magnetic phase transition. ${\vec{P}}_{S}\;$ is the reason for a change in the hybridization of the magnetic V-Cl-V bonds in such a way that an incommensurate noncollinear spiral structure can form below $T^{AFM(FM)}$. 

Similarly, we expand the isotropic exchange interaction $J_{ij}^{b}$ of the displacement u of the V ions:

(4) $$-\gamma_{1}^{*}\sum_{<ij>NNb}\left|{\vec{P}}_{S}\right|\;{\vec{S}}_{i}\cdot {\vec{S}}_{j}-\gamma_{2}^{*}\sum_{i<ij>NNb}{\left|{\vec{P}}_{S}\right|}^{2}{\vec{S}}_{i}\cdot {\vec{S}}_{j},$$

where $\gamma_{1}^{*}=\gamma_{1}/e^{*}$, $\gamma_{2}^{*}=\gamma_{2}/{\left(e^{*}\right)}^{2}$, as $\gamma_{1}$ and $\gamma_{2}$ are the first and second derivatives, respectively, of the isotropic exchange magnetic interaction $J_{ij}^{b}$ with respect to the polar equilibrium displacements u.

This means that in the Hamiltonian of the system, describing both magnetic and ferroelectric properties, an additional term appears: $-\lambda^{*}\sum_{<ij>NNb}\left({\vec{P}}_{S}\times {\vec{e}}_{ij}^{b}\right)\cdot \left[{\vec{S}}_{i}\times {\vec{S}}_{j}\right]$. Comparing this term with conventional DM interaction, we can conclude that the spontaneous polarization induces DM interaction with the vector ${\vec{D}}_{ind\;ij}^{b}=\lambda^{*}({\vec{P}}_{S}\times {\vec{e}}_{ij}^{b})$. We can define a temperature-dependent DM vector ${\vec{D}}_{eff\;ij}^{b}={\vec{D}}_{\;ij}^{b}+{\vec{D}}_{ind\;ij}^{b}$. Its value increases below $T^{AFM(FM)}$ with decreasing temperature. This vector can be considered as the induced DM interaction resulting from the emerging spontaneous polarization. In a similar manner, the terms in Equation (4) make the isotropic interaction temperature dependent, $J_{eff}^{b}=J^{b}+\gamma_{1}^{*}\left|P_{S}\right|+\gamma_{2}^{*}{\left|P_{S}\right|}^{2}$. The last term in Equation (3) and the expressions in (4) naturally define the relationship between the two systems—the spin and ferroelectric ones, explicitly specifying the ME interaction. The Hamiltonian describing the possible ME interactions in the VOCl_2_ monolayer is as follows:

(5) $$H_{me}=-\lambda^{*}\sum_{<ij>NNb}\left({\vec{P}}_{S}\times {\vec{e}}_{ij}^{b}\right)\cdot \left({\vec{S}}_{i}\times {\vec{S}}_{j}\right)-\gamma_{1}^{*}\sum_{<ij>NNb}\left|{\vec{P}}_{S}\right|\left({\vec{S}}_{i}\cdot {\vec{S}}_{j}\right)-\gamma_{2}^{*}\sum_{<ij>NNb}{\left|{\vec{P}}_{S}\right|}^{2}\left({\vec{S}}_{i}\cdot {\vec{S}}_{l}\right).$$

This Hamiltonian describes the simultaneous action of two types of ME couplings. The last term in Equation (5) defines the ME coupling characteristic of Type I MFs. The coupling is quadratic in spins and pseudo-spin operators and can be expressed as follows: ${-\gamma }_{2}^{*}\sum_{klij}\left({\vec{B}}_{k}\cdot {\vec{B}}_{l}\right)({\vec{S}}_{i}\cdot {\vec{S}}_{j})$. The first term in the Hamiltonian defines the antisymmetric ME interaction, which, in the pseudo-spin representation, takes the form $-\lambda^{*}\sum_{kij}\left({\vec{B}}_{k}\times {\vec{e}}_{ij}\right)\cdot \left({\vec{S}}_{i}\times {\vec{S}}_{j}\right)$. The second term introduces a magnetostriction mechanism, which, in the pseudo-spin representation, is linear in pseudo-spin operators and quadratic in spins, i.e., $-\gamma_{1}^{*}\sum_{ijk}B_{i}^{x}\left({\vec{S}}_{i}\cdot {\vec{S}}_{j}\right)$. The last two interactions are characteristic of Type II MFs. This implies that in the monolayer VOCl_2_, properties of two types of MFs—proper and improper—are combined. The terms in Equation (5) play a feedback role. The spontaneous polarization induces an antisymmetric magnetic interaction of the DM type and magnetostriction effects, responsible for the appearance of the cycloidal spiral in the (ab) plane (or weak FM). Additionally, the magnetization can be manipulated with changes in polarization, both in magnitude and direction. On the other hand, this coupling, through magnetization, determines the influence of magnetic ordering on the values of $P_{S}$. 

Based on the above analysis, the Hamiltonian describing the MF properties of the VOCl_2_ monolayer has the following form:

(6) $$H=H_{e}+H_{m}+H_{me}.$$

For the theoretical calculations let us introduce the following components:

(7) $${S_{i}}^{\pm }=\frac{1}{\sqrt{2}}\left({S_{i}}^{x}\pm {iS_{i}}^{y}\right);\;{S_{i}}^{z}={S_{i}}^{z};\;{B_{i}}^{\pm }=\frac{1}{\sqrt{2}}\left({B_{i}}^{y}\pm {{iB}_{i}}^{z}\right);\;{B_{i}}^{x}={B_{i}}^{x}$$

Using (7) and comparing the first two terms of Equation (1) with their counterparts in Equation (5), the Hamiltonian of the TIM can be written as follows:

(8) $$H_{e}=-\Omega^{eff}\sum_{i}B_{i}^{y}-\sum_{ij}J_{ij}^{\prime eff}B_{i}^{x}B_{j}^{x},$$

where:

(9) $$\Omega^{eff}=\left\{\begin{array}{c} \Omega \;for\;T\geq T^{AFM(FM)}\\ \Omega +\lambda^{*}\sum_{kl}\left(<S_{k}^{z}S_{l}^{+}>+{<S}_{l}^{z}S_{k}^{-}>\right)\;for\;T<T^{AFM(FM)} \end{array}\right.;$$

(10) $$J_{ij}^{\prime eff}=\left\{\begin{array}{c} J_{ij}^{\prime }\;for\;T\geq T^{AFM(FM)}\\ J_{ij}^{\prime }+2\gamma_{2}^{*}\sum_{kl}\left(<S_{k}^{-}S_{l}^{+}>+{<S}_{l}^{z}><S_{k}^{z}>\right)\;for\;T<T^{AFM(FM)} \end{array}\right..$$

The last two expressions determine the influence of magnetic ordering for $T<T^{AFM(FM)}$ on the frequency rate Ω and pseudo-spin interaction $J^{\prime }$. They become temperature dependent.

From Equation (1), it is evident that the FE phase is characterized by two non-zero mean values, ${<B}_{i}^{x}>$ and ${<B}_{i}^{y}>$. For convenience, we will transition to a single order parameter, ${<B}_{i}^{x}>$ (which defines polarization). To achieve this, we will rotate the local coordinate system around the z-axis by an angle Θ. This rotation is chosen so that, at every temperature, the condition ${<B}_{i}^{y}>=0$ holds, meaning ${<[B}_{i}^{y};H]>=0$. This condition provides the opportunity to determine the angle Θ:

(11) $$sin\Theta_{i}=\frac{\Omega_{eff}}{\sum_{j}J_{ij}^{\prime eff}<B_{j}^{x}>},$$

where ${<B}_{i}^{x}>$ represents the mean value of the operators of the dipole moment.

To investigate the pseudo-spin system, we define the following Green’s function:

(12) $${G_{ij}^{e}=\ll B_{i}^{+};B_{j}^{-}\gg }_{E}.$$

Using the equation of motion for the Green’s function:

(13) $$EG_{ij}^{e}=\left(\frac{i}{2\pi }\right)\langle \left[B_{i}^{+};B_{j}^{-}\right]\rangle +\langle \langle \left[B_{i}^{+};H\right];B_{j}^{-}\rangle \rangle ,$$

we obtain:

(14) $$<B_{i}^{x}>=\frac{1}{2}th\left(\frac{\omega_{i}}{2k_{B}T}\right){cos\Theta }_{i},$$

where $\omega_{i}$ is the energy of the pseudo-spin excitation and is determined by the pole of the Green’s function from Equation (13):

(15) $$\omega_{i}=2\Omega^{eff}sin\Theta_{i}+\sum_{j}J_{ij}^{\prime eff}<B_{j}^{x}>cos\Theta_{j}{cos\Theta }_{i}.$$

Thus, for the spontaneous polarization, we obtain:

(16) $$P_{S}=\frac{1}{N}\sum_{i}<B_{i}^{x}>=\frac{1}{2N}\sum_{i}th\left(\frac{\omega_{i}}{2k_{B}T}\right){cos\Theta }_{i}.$$

For the description of the magnetic subsystem and the calculation of magnetization, we define the following system of spin-retarded Green’s functions in the energy presentation:

$${\ll S_{i}^{+};S_{j}^{-}\gg }_{E};{\ll S_{i}^{-};S_{j}^{+}\gg }_{E};{\ll S_{i}^{-};S_{j}^{-}\gg }_{E};$$

(17) $${\ll S_{i}^{+};S_{j}^{+}\gg }_{E};{\ll S_{i}^{z};S_{j}^{+}\gg }_{E};{\ll S_{i}^{z};S_{j}^{-}\gg }_{E}.$$

Using the equation of motion (13), we calculate the Green’s functions in the Random Phase Approximation (RPA). Their analytical expressions are provided in Appendix A.

The magnetization is determined by:

(18) $$M=\frac{1}{N}\sum_{i}<S_{i}^{z}>.$$

as $<S_{i}^{z}>$ is calculated from the expression in [44]:

(19) $$<S_{i}^{z}>=\left(S+0.5\right)coth\left[\left(S+0.5\right)\beta \phi_{i}\right]-0.5coth\left[0.5\beta \phi_{i}\right],$$

where $\beta =k_{B}T$, $\phi_{i}=\frac{1}{N}\sum_{j}\phi_{ij}$. The analytical expression for $\phi_{ij}$ is given in the Appendix A.

For calculating the correlation functions, we use the spectral theorem [45]:

$$<S_{i}^{-}S_{j}^{+}>=\frac{1}{2\pi }\left[\Delta_{ij}\left(E_{1ij}\right)+\Delta_{ij}\left(E_{2ij}\right)\right]<S_{i}^{z}>\delta_{ij};$$

$$<S_{i}^{+}S_{j}^{-}>=-\frac{1}{2\pi }\left[\Psi_{ij}\left(E_{1ij}\right)+\Psi_{ij}\left(E_{2ij}\right)\right]<S_{i}^{z}>\delta_{ij};$$

(20) $$<S_{i}^{-}S_{j}^{-}>=-<S_{i}^{+}S_{j}^{+}>=\frac{1}{2\pi }\left[\Phi_{ij}\left(E_{1ij}\right)+\Phi_{ij}\left(E_{2ij}\right)\right]<S_{i}^{z}>\delta_{ij};$$

$$<S_{i}^{z}S_{j}^{-}>=<S_{i}^{z}S_{j}^{+}>=\frac{8D^{eff}}{2\pi }\left[\Phi_{ij}\left(E_{1ij}\right)+\Phi_{ij}\left(E_{2ij}\right)\right]<S_{i}^{z}>\delta_{ij}=\frac{8d_{eff}^{z}}{\beta^{\prime }}<S_{i}^{+}S_{j}^{+}>.$$

where: 

$\Delta_{ij}\left(E\right)=\frac{\left(E+\alpha^{\prime }-\alpha^{\prime \prime }\right)}{\left[2E-\left(E_{1ij}+E_{2ij}\right)\right]}\times {\left(e^{\beta E}-1\right)}^{-1}$;

$\Psi_{ij}\left(E\right)=\frac{\left(E-\alpha^{\prime }-\alpha^{\prime \prime }\right)}{\left[2E-\left(E_{1ij}+E_{2ij}\right)\right]}\times {\left(e^{\beta E}-1\right)}^{-1}$;

$\Phi_{ij}\left(E\right)=\frac{\beta^{\prime }}{\left[2E-\left(E_{1ij}+E_{2ij}\right)\right]}\times {\left(e^{\beta E}-1\right)}^{-1}$.

$E_{1,2ij}\;$ is the magnetic energy between two local spins at i and j sites and is determined by the poles of the Green’s functions (17):

(21) $$E_{1,2ij}=\alpha^{\prime \prime }\pm \sqrt{{\left(\alpha^{\prime }\right)}^{2}-{\left(\beta^{\prime }\right)}^{2}},$$

as $\alpha^{\prime }$, $\alpha^{\prime \prime }$ and $\beta^{\prime }$ are given in Appendix A.

Within this study, we will compute the relative dielectric permittivity $\varepsilon \left(\vec{k},\;E\right)$ based on the following equation [46]:

(22) $$\left[{\left(\frac{\Lambda }{\varepsilon \left(\vec{k},E\right)-1}\right)}_{\alpha \beta }+\Lambda \frac{k_{\alpha }k_{\beta }}{k^{2}}\right]G^{\beta \gamma }\left(E\right)=\delta_{\alpha \gamma };$$

with $\Lambda =4\pi Z^{2}/\nu$ [47]. Specifically, $\varepsilon \left(\;E\right)$ of the system is related to the longitudinal anticommutator Green’s function $G^{xx}={\ll B}_{i}^{x};B_{j}^{x}\gg$, which we calculate using the Tserkovnikov method [48]. For more information on the calculation of the Green’s function, see [47].

## 3. Numerical Calculations and Discussion

For the numerical calculations of the MF characteristics of the VOCl_2_ monolayer, we will use the following model parameters:

1. For the FE subsystem: As mentioned in Section 1, the depth of the double-well potential in VOX_2_, where X = Cl, Br, I, is comparable to that of “classical” ferroelectrics with the ABO_3_ structural formula. For X = Cl, Br, I, DFT calculations determine values of 0.18, 0.132, and 0.12 eV, respectively. By comparing these values with the depth of the double-well potential for typical ferroelectrics such as BiFeO_3_, PbTiO_3_ and LiNbO_3_ at the ferroelectric phase transition temperature for VOX_2_, we approximate the following values: $T^{FE}\left(VO{Cl}_{2}\right)\sim 1026\;K$, $T^{FE}\left(VO{Br}_{2}\right)\sim 750\;K$ and $T^{FE}\left(VOI_{2}\right)\sim 690\;K$. This allows us, based on the TIM to determine the value of the exchange interaction between pseudo-spins $J^{\prime }$ and the flipping rate Ω, following [32]: $J^{\prime }=350.6\;meV,$ Ω = 0.9 meV for VOCl_2_; $J^{\prime }=256.3\;meV$, Ω = 1.23 meV for VOBr_2_ and $J^{\prime }=227.21\;meV$, Ω = 1.34 meV for VOI_2_;
2. For the magnetic subsystem we obtain: $J^{b(y)}=-21.83\;meV$, $J^{a(x)}=0.39\;meV$, $J^{ab}=0.62\;meV$, $d^{z}=0.19\;meV$, $K^{c(z)}=0.11\;meV$, $K^{b(y)}=0.025\;meV$ for $VO{Cl}_{2}$; $J^{b(y)}=-11.79\;meV$, $J^{a(x)}=0.67\;meV$, $J^{ab}=0.83\;meV$, $d^{z}=0.37\;meV$, $K^{c(z)}=0.22\;meV$, $K^{b(y)}=0.054\;emV$ for $VO{Br}_{2}$ and $J^{b(y)}=0.69\;meV$, $J^{a(x)}=2.15\;meV$, $J^{ab}=0.72\;meV$, $d^{z}=0.89\;meV$, $K^{c(z)}=0.54\;meV$, $K^{b(y)}=0.11\;emV$ for $VOI_{2}$. The data are sourced from the following articles [18,19,29,49] and their supplemental materials;
3. ME coupling constants: for the ME interaction constants, we use the following values: $\gamma_{1}^{*}=13.93\;meV$, $\gamma_{2}^{*}=0.55\;meV$ and $\lambda^{*}=0.98\;meV$. The method for obtaining these values is illustrated in Appendix B.

**Figure 4:**
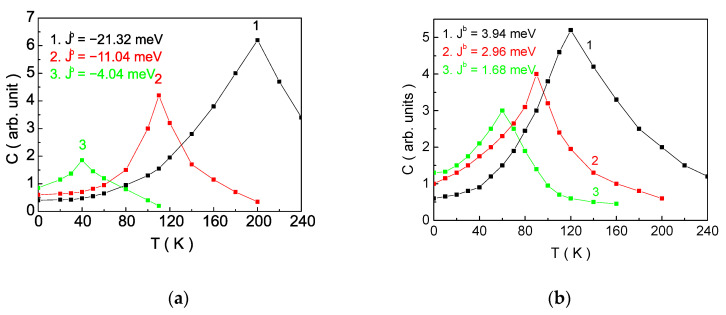
Dependence of the specific heat capacity *C* on temperature for different values of *J^b^*: (**a**) for AFM spin ordering: 1/ *J^b^* = −21.23 meV, 2/ *J^b^* = −11.04 meV, 3/ *J^b^* = −4.04; (**b**) for FM spin ordering: 1/ *J^b^* = 3.94 meV, 2/ *J^b^* = 2.96 meV, 3/ *J^b^* = 1.68 meV for the VOCl_2_ model parameters.

Figure 4 illustrates the temperature dependence of the specific heat capacity C for various values of the exchange interaction $J^{b}$. Within presented model, it is calculated using the formula $C=\frac{d<H>}{dT}$, where *H* is defined by Equation (6). From a theoretical point of view, when calculating <H>, average values of products of the following spin operators are obtained: ${<S}_{i}^{z}S_{j}^{z}>$, $<S_{i}^{z}S_{j}^{\alpha }>$ and $<S_{i}^{\alpha }S_{j}^{\beta }>$, where α,β=+,−. The longitudinal correlation function ${<S}_{i}^{z}S_{j}^{z}>$, is decoupled as $<S_{i}^{z}S_{j}^{z}>\sim <S_{i}^{z}{><S}_{j}^{z}>$, and the remaining calculations are performed using the spectral theorem (see Appendix A). This allows us to perform calculations beyond the method of the RPA. For all curves, regardless of the value of $J^{b}$, a peak is observed in the C(T) dependence. As the absolute value of the exchange interaction along the b-axis $J^{b}$ increases, the peak shifts towards higher temperature values. This peak indicates the presence of a magnetic phase transition from paramagnetic (PM) to AFM state (Figure 4a) or from PM to FM state (Figure 4b). The numerical calculations are in accordance with theoretical results obtained through Monte Carlo simulations in [18,29,49] and provide evidence for the adequacy of our model and calculation method.

**Figure 5:**
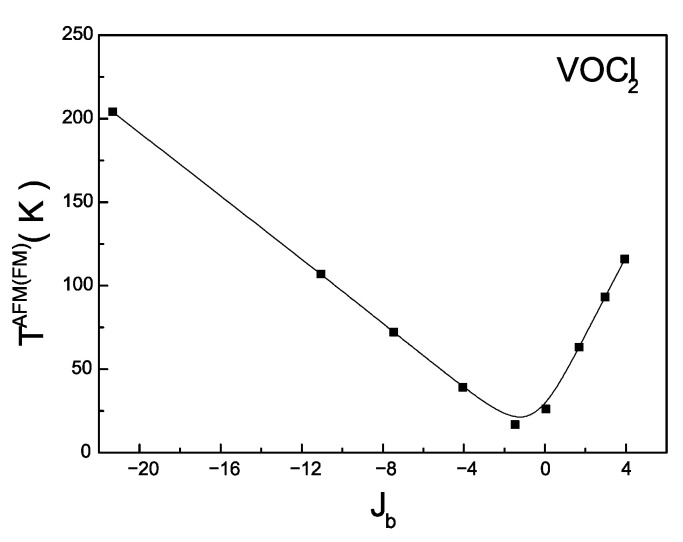
Dependence of the temperature of the magnetic phase transition *T^AFM^*^(*FM*)^ on the exchange interaction along the *b*-axis *J^b^*.

Figure 5 depicts the dependence of the magnetic phase transition temperature on the magnitude of the magnetic interaction along the b-axis $J^{b}$. The calculations are performed with model parameters for a VOCl_2_ monolayer. As the magnitude of the AFM interaction $J^{b}$ decreases, the Neel temperature decreases. This models a process in which the direct AFM exchange interaction decreases, leading to the superexchange FM interaction between V ions, assisted by Cl ions, and starts to increasingly compete with the direct interaction. As a result, the effective magnetic interaction between the magnetic moments of V ions decreases, leading to a reduction in the Neel temperature. The curve reaches a minimum at the temperature of the phase transition, which can be interpreted as a transition from AFM to FM ordering. Numerical calculations show that this occurs at a value of the effective magnetic interaction $J^{b}=-1.44\;meV$ (the calculation is made assuming that $J^{a}$ and $J^{ab}$ do not change their values). By changing the sign of $J^{b}$, we assume that the direct exchange interaction is smaller (or absent), and FM ordering is observed in the system. In this case, with an increase in $J^{b}$, the temperature of the magnetic phase transition also increases.

In VOX_2_ monolayer, replacing the halogen ion Cl with Br or I results in a significant increase in the lattice constant along the b-axis (3.380 Å for Cl, 3.585 Å  for Br and 3.956 Å for I [19]). As the exchange interactions are sensitive to the distance between interacting spins, this will lead to a rapid decrease in the direct exchange interaction between the magnetic moments of V ions, causing the effective coupling along the b-axis to become FM. This is a consequence of the FM superexchange interaction in the V-X-V bond. Such behaviour in VOX_2_ monolayers has been predicted by DFT calculations [36] under applied tensile stress along the b-axis (Supplemental Materials in [36]). In our opinion, this is the reason for observing AFM ordering in VOCl_2_ and VOBr_2_ monolayers, while VOI_2_ monolayer exhibits FM ordering. Substituting Cl with Br induces lattice tensile effects (since the Cl ion has a smaller radius than Br by 16%) along the b-axis, reducing the overlap between $d_{xy}$ orbitals and decreasing the magnitude of the direct exchange interaction (in absolute values). This decreases the value of $\left|J^{b}\right|$, leading to a reduction in the Neel temperature (Figure 4a and Figure 5) [18]. If Cl is replaced with I, the lattice tensile effect will be even greater (as the Cl ion has a smaller radius than I by 34%), and there will be no overlap of $d_{xy}$ orbitals between V ions along the b-axis. In this case, due to the superexchange interaction, there is FM ordering in the layer [29]. Numerical calculations with the above defined model parameters, within our model, predict the following temperatures for the magnetic phase transition: $T^{AFM}=114\;K\;$ for VOBr_2_ and $T^{FM}=26\;K\;$ for VOI_2_. Our obtained results are in good agreement with [18,29].

**Figure 6:**
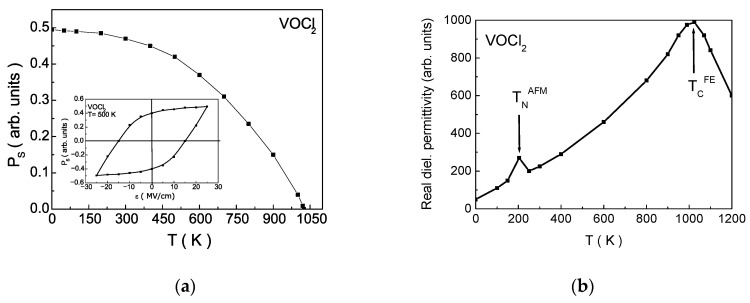
(**a**) Dependence of the spontaneous polarization *P_S_* on temperature T, (inset: dependence of *P_S_* on the applied external electric field *ε* at *T* = 500 K (hysteresis curve)); (**b**) dependence of the real part of the dielectric permittivity on temperature *T*.

**Figure A1:**
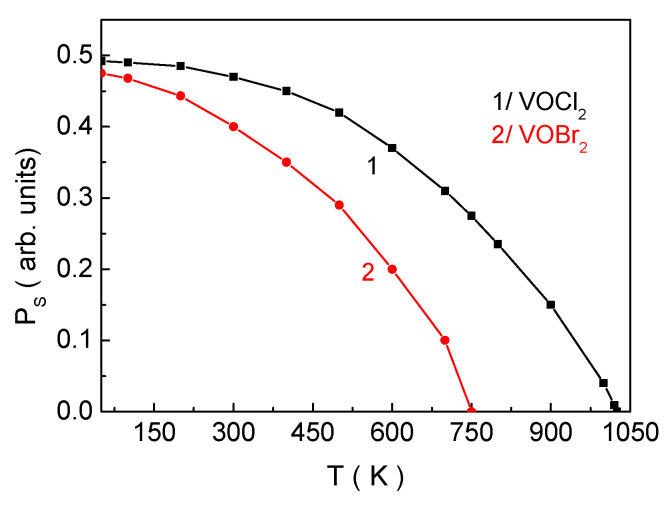
Temperature dependence of the spontaneous polarization *P_S_* for: 1/ VOCl_2_ and 2/ VOBr_2_.

Figure 6a presents the temperature dependence of the spontaneous polarization $P_{S}$. Numerical calculations reveal the presence of a hysteresis curve (inset in Figure 6a). The obtained results demonstrate the existence of the FE phase. It is evident that the temperature of the FE phase transition $T^{FE}$ is significantly higher than the temperature of the AFM phase transition in VOCl_2_ (the temperature dependence of $P_{S}$ for VOBr_2_ is given in Appendix C; Figure A1). These curves indicate the presence of a multiferroic phase with characteristics of type I MFs. The coercive field value is $15\frac{MV}{cm}$, comparable to that of typical ferroelectrics with the structural formula ABO_3_. The obtained numerical value for the coercive field is consistent with calculations in [19,49], proving that the use of TIM in a pseudo-spin representation to describe the FE system is justified and provides an adequate depiction of the processes. The observed kink in the real part of the dielectric permeability (Figure 6b) around the temperature of the magnetic phase transition is evidence of the presence of ME interaction and confirms that these compounds are MF.

**Figure 7:**
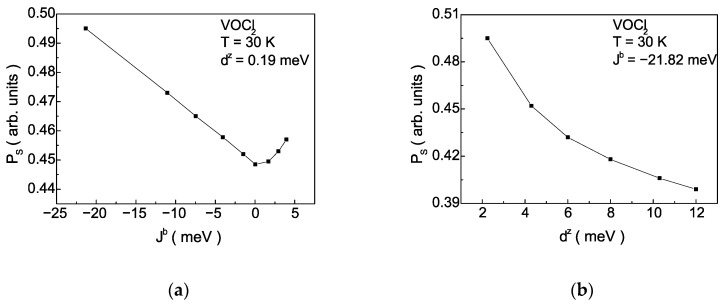
Dependence of the spontaneous polarization *P_S_* on: (**a**) the exchange magnetic interaction *J^b^* along the *b*-axis; (**b**) the magnitude of the DM vector *d^z^* of the antisymmetric interaction between neighboring spins along the *b*-axis for VOCl_2_ monolayer at *T* = 30 K.

Figure 7a depicts the dependence of the magnitude of the spontaneous polarization $P_{S}$ on the magnitude and sign of the exchange interaction $J^{b}$ along the b-axis. As the magnitude of $\left|J^{b}\right|$, decreases, in the case of AFM ordering of spins in the monolayer, the value of $P_{S}$ decreases by about 10%. In the case of FM ordering of spins in the monolayer, the polarization increases with an increase in the value of $J^{b}$. Such a dependence is theoretically predicted in DFT modelling of processes under tensile strains along the b-axis [36]. In monolayers of VOCl_2_ and VOBr_2_, a decrease in $P_{S}$ is observed when Cl is replaced with Br [18]. By this substitution, due to the difference in the anionic radii, the lattice constant along the b-axis will increase, leading to weaker overlap between the $d_{xy}$ orbitals between V ions and a decrease the value of direct exchange interaction. With constant values of the other model parameters of VOCl_2_, the change in polarization is a consequence only of magnetoelastic interaction, described by the Hamiltonian $H_{me}$ from Equation (6). For VOI_2_, it has been calculated [29] that $P_{S}$ is smaller compared to that in VOCl_2_ and VOBr_2_. Considering the model values for $J^{b}$ for the three compounds, this behaviour aligns with our calculations.

**Figure 8:**
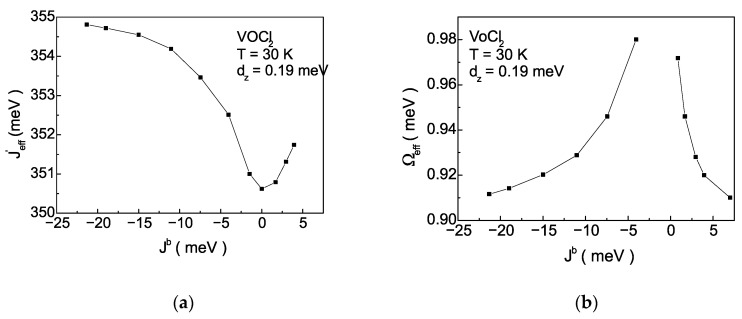
Dependence on the exchange magnetic interaction $J^{b}$ along the b-axis of: (**a**) the effective pseudo-spin interaction ${\;J}_{eff\;}^{\prime }$; (**b**) the effective flipping rate Ω*_eff_* at T=30 K for VOCl_2_.

The explanation from a microscopic point of view is related to Equations (9) and (10), which illustrate the influence of the magnetic system (the $J^{b}$ interaction) on the exchange interaction between pseudo-spins $J^{\prime }$ and flipping rate Ω. These dependencies are presented in Figure 8a,b. The magnetoelastic interaction renormalizes $J^{\prime }$ and Ω, making them temperature dependent. As the magnitude of $\left|J^{b}\right|$ decreases in the case of AFM ordering of spins in the monolayer, $J_{eff}^{\prime }$ decreases, while in the case of FM ordering of spins in the monolayer, $J_{eff}^{\prime }$ increases (Figure 8a). The behaviour of Ω*_eff_* is opposite (Figure 8b). It is important to note that numerical calculations are performed at a fixed temperature. Qualitatively, this behaviour is explained as follows: the decrease in $\left|J^{b}\right|$ is the cause for the decrease in the Neel temperature of the magnetic phase transition $T^{AFM}$. Then, at a fixed temperature, the magnetization will decrease, and according to Equation (9), the value of $J_{eff\;}^{\prime }$ will reduce. With the decrease of $\left|J^{b}\right|$, the anomalous correlation functions $<S_{i}^{\alpha }S_{j}^{\alpha }>$, α=+,− will increase, because thermal fluctuations increase, leading to an increase in Ω*_eff_* according to Equation (10) and Appendix A (Figure 8b). The decrease in $J_{eff}^{\prime }$ and the increase in Ω*_eff_* within the magnetoelastic interaction will lead to a decrease in polarization at a fixed temperature [32]. This behaviour is illustrated in Figure 7a for negative values of $J^{b}$. As is well known, Ω*_eff_* determines the height of the double-well potential. This means that with the decrease in $\left|J^{b}\right|$, the height of the double-well potential will decrease too. The decrease in $\left|J^{b}\right|$, models processes of tensile strain along the b-axis and a decrease in polarization. This behaviour is theoretically predicted in [36]. Conversely, with an increase in $J^{b}$ in the case of FM ordering of spins the Curie temperature of the magnetic phase transition $T^{FM}$ increases. Then, at a fixed temperature, the magnetization will increase, and according to Equation (9), the value of $J_{eff\;}^{\prime }$ will also increase. With the increase in $J^{b}$, the value of the anomalous correlation functions $<S_{i}^{\alpha }S_{j}^{\alpha }>$, α=+,− will decrease, leading to reducing Ω*_eff_* according to Equation (10) (Figure 8b). The increase in $J_{eff}^{\prime }$ and the decrease in Ω*_eff_*, within the TIM, will lead to an increase in the polarization at a fixed temperature [32], i.e., the stabilization of the FE phase. 

Figure 7b represents the dependence of the spontaneous polarization $P_{S}$ on the value of the DM vector $d_{z}$. An increase in the antisymmetric exchange interaction leads to a decrease in the value of the spontaneous polarization. The reason for this is that with an increase in $d_{z}$, the value of the flipping rate Ω*_eff_* increases. This means that at a fixed temperature, the polarization will decrease. Our numerical calculations show that regardless of the sign of $J^{b}$, an increase in $d_{z}$ results in a decrease in polarization. The decrease in Ω*_eff_* within our model implies an increase in the height of the double-well potential. Such behavior is predicted in DFT calculations when replacing a Cl anion with an I anion, where the spontaneous polarization decreases. The I atom has a higher atomic number in the periodic table, and in the structure of VOX_2_, this corresponds to a larger value of *d_z_*(VOI_2_) compared *d_z_*(VOCl_2_). This is not the primary reason for the decrease in $P_{S}$, but this dependence unequivocally demonstrates the influence of antisymmetric magnetic interactions on the polarization subsystem in VOX_2_ monolayers.

**Figure 9:**
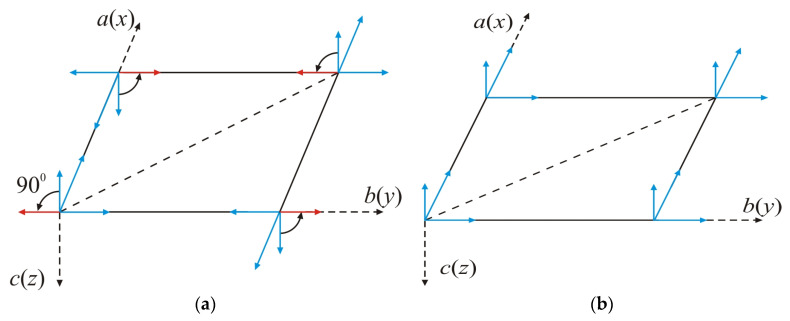
Schematic representation of (**a**) the direction of the components of V spins for AFM ordering and magnetization along the *c*-axis (blue vectors) and the direction of the components of V spins and magnetization along the *b*-axis after a SR transition without a change in the sign and magnitude of *J^b^* (red vectors); (**b**) the direction of the components of V spins for FM ordering with a change in the sign of *J^b^* without an SR transition.

Within our model, we can qualitatively analyse the possibility of a SR transition. As noted in Section 2, to observe SR transitions, it is necessary to have a term in the Hamiltonian describing the magnetic subsystem that depends on direction. This is the antisymmetric DM interaction. We showed that for VOX_2_ monolayers, due to the spontaneous polarization $P_{S}$, such an interaction is induced, with DM vector taking the following form: ${\vec{D}}_{ind\;ij}^{b}=\lambda^{*}({\vec{P}}_{S}\times {\vec{e}}_{ij}^{b})$. In this case, to minimize the total energy of the system, it is necessary for the polarization and magnetization to be perpendicular. Using Figure 2a, it is clear that the ${\vec{D}}_{ind\;ij}^{b}$ vector has components $\left[0,\;0,\;\lambda^{*}P^{a(x)}\right]$. The ${\vec{D}}_{eff\;ij}^{b}$ expressed in coordinates has the form $\left[0,\;0,d_{eff}^{z}\right],$ where $d_{eff}^{z}=d^{z}+\lambda^{*}P^{a(x)}$, i.e., it is temperature dependent. This antisymmetric interaction will create an effective field along the c(z)-axis. When the interaction energy of the magnetic moments of V ion with this effective field reaches the value of the magnetic anisotropy energy along the c(z)-axis, the z-component of the spin will begin to rotate around the a(x)-axis by 90° until it aligns along the b(y)-axis. The reason for this is that the b-axis now becomes the easy-axis of magnetization, and the z-component of the spin will reorient along the b-axis (Figure 9a). Only with this rotation the requirement that the magnetization must be always perpendicular to the polarization is satisfied. This can be achieved by changing the magnitude of ${\vec{D}}_{eff\;ij}^{b}$. If, during this rotation, the magnitude and sign of $J^{b}$ do not change, then after the rotation, the arrangement will remain AFM (red arrows in Figure 9a). Speculating, if an electric field is applied along the a(x)-axis, this could lead to a significant change in the value of $P_{S}$, triggering an SR transition while preserving AFM ordering of spins in the (ab) plane.

Our numerical calculations for the total energy of the system $E_{tot}=<H>$ for AFM ordering with magnetization along the c(z)-axis and AFM ordering with magnetization along the b(y)-axis with a change in the value of $d^{z}$ show that for values of the DM vector in the range (0÷0.59) meV, it is energetically more favorable to realize AFM ordering with magnetizatcion along the c(z)-axis. For $d^{z}>0.59\;meV$, it is more advantageous to realize AFM ordering with magnetization along the b(y)-axis. Such a transition (rotation of the easy-plane magnetization without changing the character of the magnetic ordering) has been experimentally observed in the compound BiFeO_3_ [50]. According to us, the described situation can be experimentally tested.

If only the sign of $J^{b}$ changes, leading from AFM to FM ordering, then an SR transition will not be observed because the magnetic anisotropy constant along the c(z)-axis is larger compared to that along the b(y)-axis. According to us, in this case, we will have FM ordering of spins oriented along the c(z)-axis. This will ensure a minimum of the total energy of the system, guaranteeing mutual perpendicularity of polarization and magnetization and minimizing the magnetocrystalline anisotropy (Figure 9b).

Based on the obtained numerical expressions for $J_{eff}^{b}=J^{b}+\gamma_{1}^{*}\left|P_{S}\right|+\gamma_{2}^{*}{\left|P_{S}\right|}^{2}$ and $d_{eff}^{z}=d^{z}+\lambda^{*}\left|P_{S}\right|$, it is possible to analyze the influence of ME coupling on spin interactions.

**Figure 10:**
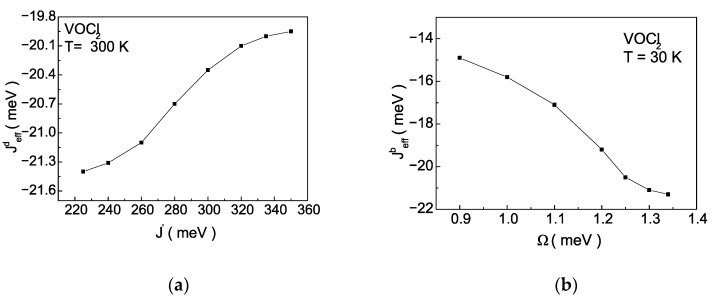
Dependence of the effective exchange interaction $J_{eff}^{b}$ along the b-axis on (**a**) pseudo-spin interaction $J^{\prime }$ and (**b**) flipping rate Ω, at T=30 K for a monolayer of VOCl_2_.

In Figure 10a,b, the dependence $J_{eff}^{b}$ on the pseudo-spin interaction $J^{\prime }$ and flipping rate Ω is presented. An increase in the value of $J^{\prime }$ leads to a decrease in the absolute value of the exchange interaction along the b-axis, while an increase in the value of Ω is the cause of an increase in $\left|J_{eff}^{b}\right|$. The range of variation of $J^{\prime }$ and Ω is within the values determined at the beginning of Section 3. In both cases, the sign of the exchange interaction $J_{eff}^{b}$ is preserved, indicating that the ME interaction does not change the character of the magnetic ordering within our model with the selected model parameters. It remains antiferromagnetic with magnetization along the c(z)-axis. According to Figure 5, the ME interaction renormalizes the temperature of the magnetic phase transition in the range from 204 K to 157 K. A qualitative explanation of the observed dependencies can be provided by the fact that the simultaneous increase in $J^{\prime }\;$ and the decrease in Ω stabilizes the ferroelectric phase, increasing the temperature of the FE phase transition $T^{FE}.$ Then, at a fixed temperature, the value of the spontaneous polarization $P_{S}$ will increase. DFT studies [36] show that applying tensile strain along the a-axis reduces $\left|J_{eff}^{b}\right|$, and with increasing tensile stress, the depth of the double-well potential increases. Within the TIM this leads to a decrease in the value of Ω and a decrease in the temperature of the magnetic phase transition. These facts are consistent with the analysis conducted by us on Figure 10a,b.

**Figure 11:**
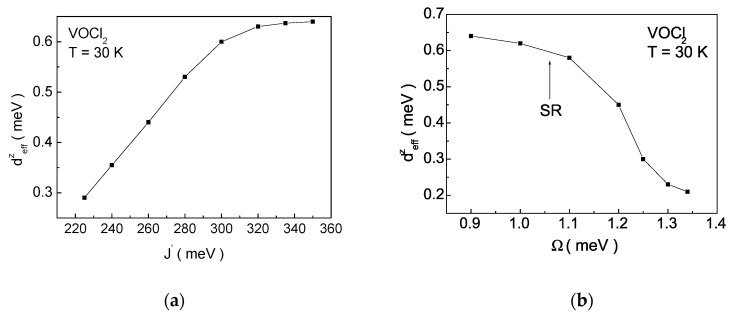
Dependence of the effective DM vector $d_{eff}^{z}$ on (**a**) the pseudo-spin interaction $J^{\prime }\;$ and (**b**) the flipping rate Ω at T=30 K for a VOCl_2_ monolayer.

Figure 11 depicts the dependence of the effective value of the DM vector $d_{eff}^{z}$ on the pseudo-spin interaction $J^{\prime }$ and the flipping rate Ω. Increasing the values of both parameters ($J^{\prime }$ and Ω) for the FE system leads to an increase (Figure 11a) and decrease (Figure 11b) in $d_{eff}^{z}$, respectively. In the multiferroic $VOX_{2}$, $d_{eff}^{z}$ significantly increases with the deepening of the double-well potential, achieved by applying tensile strain along the a-axis [36]. Magnetoelectric coupling renormalizes DM vector and influences the magnetic characteristics. With the chosen model parameters, the system can exhibit a SR transition due to ME interactions in the system (Figure 11b).

It should be noted that the lack of experimental data for VOX_2_ monolayers does not allow for the precise determination of model parameters that define the MF behaviour of this compound at the microscopic level.

From the qualitative analysis, it is clear that to observe a SR transition from AFM ordering along the c(z)-axis to FM one along the b(y)-axis, it is necessary simultaneously the value of ${\vec{D}}_{eff\;ij}^{b}\;$ to increase and the sign of $J^{b}$ to change. This is possible with increasing distance between the spins along the b-axis. Then, the direct exchange interaction between the $d_{xy}$ orbitals of V ions along this axis becomes smaller compared to the superexchange interaction in the V-X-V bond. This can occur by substituting Cl(Br) anions with I ions, resulting in a change in the nature of the interaction along the b-axis from AFM to FM, and simultaneously increasing the value of DM vector. According to our analysis, this could be experimentally demonstrated by doping, replacing Cl(Br) atoms with I atoms, i.e., VOA_2(1−*x*)_I_2*x*_, where A = Cl, Br.

In summary: In the present article, a microscopic model of the MF VOX_2_, X = Cl, Br, I, monolayers is constructed. Despite the formal violation of the “$d^{0}$ rule” in these compounds, a MF phase is observed. Based on symmetry analysis, it is justified that the possible ME interactions characterize VOX_2_ as MF with distinctive features of type I and type II. The numerical calculations demonstrate that our proposed microscopic Hamiltonian adequately describes the properties of the magnetic and electric systems. Model parameters and exchange interactions used in the calculations and analysis of the MF behaviour of the system are justified, too. Using a Peierls-type interaction between the magnetic and lattice systems, we derive the possible interaction mechanisms between the magnetic and polarization order parameters, i.e., the ME interactions. This allows us to represent analytically and graphically the influence of the exchange interaction $J^{b}$ along the b-axis on the values of the pseudo-spin interaction $J^{\prime }$ and flipping rate Ω, discussing the dependence of $P_{S}$ on these parameters. It is shown that $P_{S}$ in VOX_2_ induces an antisymmetric exchange magnetic interaction of DM type. Based on the defined Hamiltonians of the system, the possibility of a spin SR transition is discussed. It is demonstrated that the first term in the Hamiltonian describing the ME interactions (5) may be responsible for the reorientation of magnetization from the c(z)-axis to the b(y)-axis preserving AFM spin ordering. Changing the sign of the exchange interaction along the *b*-axis changes the character of the magnetic ordering from AFM to FM but does not lead to an SR transition. It is shown that the last two terms in the Hamiltonian of the ME interaction reduce the value of $J^{b}$ in absolute terms but do not change its sign. Only simultaneous changes in $d_{eff}^{z}\;$ and $J^{b}$ can induce a SR transition with a change in the character and direction of the magnetic ordering. The presented numerical calculations qualitatively coincide with conclusions drawn from DFT calculations [18,19,29,36]. Unfortunately, the lack of experimental results does not allow testing the presented theoretical model, nor to precisely determine the values of the interaction constants in the FE and magnetic subsystems and between them. According to us, the presented theoretical model can be tested through doping by substituting Cl(Br) atoms with I atoms, i.e., VOA_2(1−*x*)_I_2*x*_, where A = Cl, Br.

In summary, numerical calculations and discussions provide a detailed understanding of the multiferroic characteristics of VOX_2_, X = Cl, Br, I, monolayers, shedding light on the interplay between FE and magnetic properties. The results offer valuable insights for future experimental validations and further exploration of MF materials.

## Data Availability

Derived data supporting the findings of this study are available from the corresponding author upon reasonable request.

## Appendix A

The calculated Green’s functions:

(A1) $${\ll S_{i}^{+};S_{j}^{-}\gg }_{E}=-\frac{i}{\pi }\times \frac{\left(E+\alpha^{\prime }-\alpha^{\prime \prime }\right)<S_{i}^{z}>\delta_{ij}}{\left(E+\alpha^{\prime }-\alpha^{\prime \prime }\right)\left(E-\alpha^{\prime }-\alpha^{\prime \prime }\right)+{\left(\beta^{\prime }\right)}^{2}};$$

(A2) $${\ll S_{i}^{-};S_{j}^{+}\gg }_{E}=\frac{i}{\pi }\times \frac{\left(E-\alpha^{\prime }-\alpha^{\prime \prime }\right)<S_{i}^{z}>\delta_{ij}}{\left(E+\alpha^{\prime }-\alpha^{\prime \prime }\right)\left(E-\alpha^{\prime }-\alpha^{\prime \prime }\right)+{\left(\beta^{\prime }\right)}^{2}};$$

(A3) $${\ll S_{i}^{-};S_{j}^{-}\gg }_{E}={\ll S_{i}^{+};S_{j}^{+}\gg }_{E}=\frac{i}{\pi }\times \frac{\beta^{\prime }<S_{i}^{z}>\delta_{ij}}{\left(E+\alpha^{\prime }-\alpha^{\prime \prime }\right)\left(E-\alpha^{\prime }-\alpha^{\prime \prime }\right)+{\left(\beta^{\prime }\right)}^{2}};$$

(A4) $${\ll S_{i}^{z};S_{j}^{+}\gg }_{E}={\ll S_{i}^{z};S_{j}^{-}\gg }_{E}=\frac{i}{\pi }\times \frac{8D^{eff}<S_{i}^{z}>\delta_{ij}}{\left(E+\alpha^{\prime }-\alpha^{\prime \prime }\right)\left(E-\alpha^{\prime }-\alpha^{\prime \prime }\right)+{\left(\beta^{\prime }\right)}^{2}}=\frac{8D^{eff}}{\beta^{\prime }}{\ll S_{i}^{+};S_{j}^{+}\gg }_{E}.$$

where:

(A5) $$\alpha^{\prime }=2\sum_{l}J_{effil}^{b}{<S}_{l}^{z}>\delta_{ij}+J_{effil}^{b}{<S}_{i}^{z}>\delta_{ij}+2\sum_{\xi l}J_{il}^{\xi }{<S}_{l}^{z}>\delta_{ij}+J_{il}^{\xi }{<S}_{i}^{z}>\delta_{ij}+\;+K^{c}{<S}_{l}^{z}>\delta_{ij};$$

(A6) $$\alpha^{\prime \prime }=2\sum_{l}{D_{efflj}^{b}<S}_{l}^{z}>\delta_{ij}+g\mu_{B}h;$$

(A7) $$\beta^{\prime }=-\sum_{l,}J_{effil}^{b}{<S}_{l}^{z}>\delta_{ij}-\frac{1}{2}\sum_{l}J_{effil}^{b}{<S}_{l}^{z}>\delta_{ij}-\sum_{\xi ,l,}J_{il}^{\xi }{<S}_{l}^{z}>\delta_{ij}-\frac{1}{2}\sum_{\xi ,l}J_{il}^{\xi }{<S}_{l}^{z}>\delta_{ij},$$

as: ξ=a, c.

$$\phi_{ij}=2\sum_{l}J_{effil}^{b}{<S}_{l}^{z}>\delta_{ij}-J_{effij}^{b}n_{ij}^{\pm }-J_{effij}^{b}n_{ij}^{\mp }-J_{effij}^{b}{<S}_{i}^{z}>\delta_{ij}+$$

$$+2\sum_{\xi ,l}J_{il}^{\xi }{<S}_{l}^{z}>\delta_{ij}-J_{ij}^{\xi }n_{ij}^{+-}-J_{ij}^{\xi }n_{ij}^{-+}-J_{ij}^{\xi }{<S}_{i}^{z}>\delta_{ij}+$$

(A8) $$+2\sum_{l}D_{lj}^{eff}(n_{lj}^{\pm }-n_{lj}^{-+})+K^{c}({n_{lj}^{-+}-n_{lj}^{+-}+<S}_{l}^{z}>\delta_{ij}),$$

(A9) $$n_{ij}^{+-}=\frac{<S_{i}^{+}S_{j}^{-}>}{2{<S}_{i}^{z}>\delta_{ij}};n_{ij}^{-+}=\frac{<S_{i}^{-}S_{j}^{+}>}{2{<S}_{i}^{z}>\delta_{ij}}.$$

## Appendix B

Expanding the Hamiltonian describing the magnetic properties of VOX_2_ in a series of polar displacements u we obtained:

(A10) $$H_{m-ph}=-\gamma_{1}\sum_{\zeta ij}({\vec{u}}_{ij}{)}^{\zeta }{\vec{S}}_{i}\cdot {\vec{S}}_{j}-\gamma_{1}\sum_{\delta \eta ij}({\vec{u}}_{ij}{)}^{\zeta }({\vec{u}}_{ij}{)}^{\eta }{\vec{S}}_{i}\cdot {\vec{S}}_{j}-\;-\lambda_{1}\sum_{\zeta ij}{({\vec{u}}_{ij}{)}^{\zeta }\left[{\vec{e}}_{ij}\times {(\vec{S}}_{i}\times {\vec{S}}_{j})\right]}^{\zeta }$$

To calculate the polar displacements, it is necessary to consider the Hamiltonian involving the lattice elastic energy:

(A11) $$H^{el}=\frac{A}{2}\sum_{ij}{\left({\vec{u}}_{ij}\right)}^{2},$$

where A is the elastic constant.

Minimizing the system’s energy with respect to the polar displacements of the V ions, i.e., solving the equation: $\frac{\partial \langle H_{m-ph}+H_{el}\rangle }{\partial {\vec{u}}_{ij}^{\zeta }}=0$ we find an expression for the equilibrium displacement $\langle {\vec{u}}_{ij}^{\zeta }\rangle$:

(A12) $$\langle {\vec{u}}_{ij}^{\zeta }\rangle =\frac{\gamma_{1}}{D}\sum_{\zeta ij}{\left({\vec{e}}_{ij}\right)}^{\zeta }\langle {\vec{S}}_{i}{\vec{S}}_{j}\rangle +\frac{\lambda_{1}}{D}\sum_{\zeta ij}\langle {\left[{\vec{e}}_{ij}\times \left({\vec{S}}_{i}\times {\vec{S}}_{j}\right)\right]}^{\zeta }\rangle ,$$

where $D=A-\gamma_{2}\sum_{\zeta ij}({\vec{e}}_{ij}{)}^{\zeta }{\vec{S}}_{i}\cdot {\vec{S}}_{j}$.

Taking into account that $P_{S}=e^{*}<u>$ and that in [19] $e^{*}$ is calculated, (i.e., $e^{*}=9.3e$), where e is the elementary electric charge, for <u>, we obtain: $<u>=\frac{P_{S}}{e^{*}}$. On the other hand, at T=0 K for <u> in the absence of an external electric field, we get: $<u>\sim \frac{\gamma_{1}}{A}M^{2}$, where M is the saturation magnetization at T=0 K. If we use the mean-field theory, M can be written as follows: $M^{2}=\frac{2J^{a}+2J^{b}+4J^{ab}}{k_{B}T^{AFM(FM)}}$, i.e., $<u>\sim \frac{\gamma_{1}}{A}.\frac{\left(2J^{a}+2J^{b}+4J^{ab}\right)}{k_{B}T^{AFM(FM)}}$. Finally, to determine $\gamma_{1}$, we use the following approximate expression:

(A13) $$\gamma_{1}=\frac{AP_{S}k_{B}T^{AFM(FM)}}{e^{*}(2J^{a}+2J^{b}+4J^{ab})}.$$

Then using the values for $P_{S}$, $T^{FM}$, $J^{a}$, $J^{b}$ and $\;J^{ab}$ for VOI_2_ from [29], we determine $\gamma_{1}^{*}$.

Following the procedure described in [51], we determine the values of $\gamma_{2}^{*}$ and $\lambda^{*}.$

## Appendix C

It is clear that for a given temperature the Ps for VOBr_2_ is less compared with those for VOCl_2_. Also, the temperature of the FE phase transition for VOBr_2_ is lower than that for VOCl_2_.
